# Artificial intelligence on the identification of risk groups for osteoporosis, a general review

**DOI:** 10.1186/s12938-018-0436-1

**Published:** 2018-01-29

**Authors:** Agnaldo S. Cruz, Hertz C. Lins, Ricardo V. A. Medeiros, José M. F. Filho, Sandro G. da Silva

**Affiliations:** 1 Centro de Tecnologia, Universidade Federal do Rio Grande do Norte UFRN, Av. Salgado Filho, Natal, Brazil; 2 Laboratory of Technological Innovation in Healthcare, Federal University of Rio Grande do Norte (UFRN), Natal, Brazil

**Keywords:** Artificial intelligence, Osteoporosis, Fracture, Neural network, Computer-aided detection system

## Abstract

**Introduction:**

The goal of this paper is to present a critical review on the main systems that use artificial intelligence to identify groups at risk for osteoporosis or fractures. The systems considered for this study were those that fulfilled the following requirements: range of coverage in diagnosis, low cost and capability to identify more significant somatic factors.

**Methods:**

A bibliographic research was done in the databases, PubMed, IEEExplorer Latin American and Caribbean Center on Health Sciences Information (LILACS), Medical Literature Analysis and Retrieval System Online (MEDLINE), Cumulative Index to Nursing and Allied Health Literature (CINAHL), Scopus, Web of Science, and Science Direct searching the terms “Neural Network”, “Osteoporosis Machine Learning” and “Osteoporosis Neural Network”. Studies with titles not directly related to the research topic and older data that reported repeated strategies were excluded. The search was carried out with the descriptors in German, Spanish, French, Italian, Mandarin, Portuguese and English; but only studies written in English were found to meet the established criteria. Articles covering the period 2000–2017 were selected; however, articles prior to this period with great relevance were included in this study.

**Discussion:**

Based on the collected research, it was identified that there are several methods in the use of artificial intelligence to help the screening of risk groups of osteoporosis or fractures. However, such systems were limited to a specific ethnic group, gender or age. For future research, new challenges are presented.

**Conclusions:**

It is necessary to develop research with the unification of different databases and grouping of the various attributes and clinical factors, in order to reach a greater comprehensiveness in the identification of risk groups of osteoporosis. For this purpose, the use of any predictive tool should be performed in different populations with greater participation of male patients and inclusion of a larger age range for the ones involved. The biggest challenge is to deal with all the data complexity generated by this unification, developing evidence-based standards for the evaluation of the most significant risk factors.

## Background

Osteoporosis is an osteometabolic disease characterized by low bone mineral density (BMD) and deterioration of the microarchitecture of the bone tissue, causing an increase in bone fragility and consequently leading to an increased risk of fractures [1]. It occurs when bone mass decreases more rapidly than the body’s ability to replace it, resulting in substantial loss of bone strength [2]. It affects all bones in the body and shows no signs or symptoms until a fracture occurs. The decrease of the bone mineral density occurs from aging and fracture rates increase over the years, causing morbidity and some mortality.

## Statistics

Annually 8.9 million fractures occur worldwide resulting from osteoporosis, which means that every 3 s a person suffers an osteoporotic fracture. The most common sites with incidence of fractures include spine, forearm, proximal humerus and hip [3]. Hip fractures are those that present higher morbidity and mortality and generate the highest direct costs for health care services, increasing exponentially when they affect the elderly population. It is estimated that by 2050 the number of hip fractures in men and women aged 50–64 years in Latin America will increase by 400%. In Brazil ten million people, about one person in every seventeen, suffer from osteoporosis [3]. The aging of the population will lead to a significant increase in the occurrence of fractures, being more evident in Asia and Latin America, since there has been an increase in the incidence of osteoporotic fractures in developing countries. The prevalence of fractures was found to be 37.5% among men and 21% among women and the costs incurred because of these fractures for private health insurance companies is estimated at around 6 million dollars a year [4].

Considered by the World Health Organization—WHO, as the silent illness of the century, causing major economic and social impacts, it has become more common than high cholesterol, allergies and common cold [5].

The high cost of treatment and the difficulty in diagnosing this disease, also considering its morbid consequences, caused by fractures, requires the development of methods that allow the identification of so-called risk groups in order to implant prevention measures at associated fractures. Measures such as these are essential for maintaining health, quality of life and independence of the population.

## Diagnostic methods

The WHO adopts as method for osteoporosis diagnosis the measurement of Bone Mineral Density. The degree of decrease is characterized by the amount of bone mineral in a particular area of the bone. This value is compared with the BMD of a population of young adults of the same gender, in the relation between these two values we have a number of standard deviation (SD). This difference between the current bone mass of the patient examined and the mass of the Population of young adults of the same gender is called T-score. According to WHO, osteoporosis is diagnosed when the T-score is more than − 2.5 SD (see Table 1).

**Table 1:** Criteria for the diagnosis of osteoporosis—T score. WHO 1994

Regular	Equal or bigger than − 1.0
Osteopenia	Between − 1.0 and − 2.5
Osteoporosis	Equal or smaller than − 2.5
Severe osteoporosis	Equal or smaller than − 2.5 with fracture due to fragility

Although other skeletal abnormalities contribute to the diagnosis, BMD measurement is used as the main factor for the determination of osteoporosis in a patient. Many techniques are available for BMD assessment, these are applied in different locations including those where osteoporotic fractures predominate.

Ultrasonography, computed tomography, radiography and magnetic resonance imaging are examples of techniques for assessing BMD and fracture risk. The absorptiometry of single and double X-ray is a method of evaluating the mineral content of the whole skeleton or a specific place, especially the most vulnerable to the occurrence of fractures. With this method, we have a more accurate density than a real volumetric density, since the scanning is two-dimensional.

Dual-energy x-ray absorptiometry (DXA) is considered the “gold standard” because it is the most developed, technically the most validated and has good performance characteristics for predicting fractures. The disadvantage of this method is that it emits ionizing radiation.

Quantitative ultrasonography (QUS) is another method, not diagnostic, since it can not provide criteria for this, but rather as an evaluation of fracture risks. This method consists of calculating the broadband ultrasound attenuation and the velocity of sound, usually measured at the patient’s heel. This method does not involve emission of ionizing radiation, it is non-invasive, it has an affordable cost and it can be used for evaluations in larger populations, but its performance is less satisfactory.

Another frequently used method for diagnosis of osteoporosis is the visual inspection of simple radiographs of sites such as metacarpal, distal phalanges, and the distal forearm. The risk assessment can be estimated using quantitative techniques, such as estimating the cortical width of the second, third and fourth metacarpals. The most used indices are the ratio of cortical width to total width or cortical area in relation to the total cross-sectional area [6]. This method has been used for many years but as assessment of fracture risk, it has been validated only in prospective studies.

Magnetic resonance imaging (MRI) is a method that, while not providing information on bone mineral density, provides some resolution of the internal structure of spongy bone [7]. Due to its high cost and complexity, MRI is currently used as an investigation procedure.

Finally, we have the computerized quantitative tomography (TQC), which also uses X-ray emission. The difference is that it is possible to capture high definition images through transverse radiographs, the images are processed by a computer forming a chain of photographs detailing the part studied. Some disadvantages in this technique are high radiation exposure and high cost when compared to DXA.

## Total costs with osteoporosis

Performing a total cost survey on osteoporosis involves aspects that are difficult to measure, such as acute hospital care, prolonged use of medications and loss of working days for those caring for the patient (see Table 2). There is great variability in methods of economic evaluation studies that address osteoporosis. Since the same instruments are not used for cost estimates, it is difficult to make direct international comparisons, even comparisons between countries, because these estimates are based on country-specific aspects such as demography, epidemiology, economics, health system and health service; Which involve health care that differ widely in treatment standards. To exemplify, in the United Kingdom, the average length of hospital stay after a fracture is 30 days, differently from Sweden which is 15 days [1]. In Portugal hip fracture treatment is treated conservatively, whereas in other countries in Europe the treatment is mostly surgical [8].

**Table 2:** Costs with treatment of fractures from the SUS perspective in Brazil ($). SUS 1994

Kind of fracture	Spine	Hips	Fists	Shoulder
Total cost	$11,933.38	$6469.29	$2273.41	$2606.28
Length of hospital stay (days)	15	27	7	5
Incidence (60–69 years old) (%)	0.3	44.4	52.1	3.2

In this context of difficulties in measuring accurately the cost of this disease the conclusion is that the costs are substantial. For example, Wales and England estimated the cost of 942 million pounds per year [9], an increase of perspective due to the growth of the elderly population. In the United States, direct medical expenses with osteoporotic fractures were estimated at US$13.8 billion in 1995 [10].

Considering the length of hospital stay, incidence, treatment costs and medication, it is clear that hip fracture has the highest costs compared to other osteoporotic fractures. The increase in these costs is directly affected by age, even doubling when compared between the elderly and the young. In a worldwide projection, current hip fracture costs stand at $3.6 billion for men and $19 billion for women, by 2050 the forecast is $14 billion for men and $73 billion for women. The direct cost estimates of osteoporotic fractures do not take into account the so-called indirect costs for the economy, which involve incapacity and loss of productivity [11].

## Objective

This article aims to gather and describe in a systematic way the main techniques used to identify risk groups on osteoporosis, recognizing their challenges and trends. Techniques that applied artificial intelligence concepts, advanced algorithms, input parameters—as significant factors to categorize risk groups—were highlighted in combination with exams already performed such as QUS, DEXA, DXA, TQC, QUA and digitized images.

## Methods

**Fig. 1:**
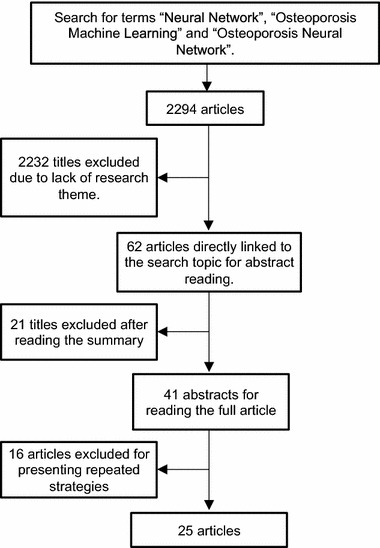
Organization chart of the methodology on the different stages of search

For this review, a bibliographic search has been conducted using the online databases PubMed—US National Library of Medicine National Institute of Health, IEEE Explorer—Digital Library and Science Direct, Latin American and Caribbean Center on Health Sciences Information (LILACS), Medical Literature Analysis and Retrieval System Online (MEDLINE), Cumulative Index to Nursing and Allied Health Literature (CINAHL), Scopus, Web of Science, for articles published between January 2000 and May 2017. Some previously published papers have been used in this review DUE to its high index of citations and relevance in relation to the theme. The search restricted to the terms “Neural Network”, “Osteoporosis Machine Learning” and “Osteoporosis Neural Network”. 2294 articles were found and 2232 titles that were not directly related to the research topic were excluded. A third screening was performed after reading the abstracts, applying criteria such as number of citations, method used for data processing, study groups (men, women, race, age group), number of individuals used in the research and quantitative variables of these items have been removed. Of the 41 selected for complete reading, 16 were excluded because they presented repeated strategies and a lower index of citations, leaving 25 more relevant articles (Fig. 1).

## Inclusion and exclusion criteria

In order to gather all possible techniques for the identification of so-called risk groups for osteoporotic fractures, we selected the most recent articles dealing with the systematic, randomized multicenter study, cohort studies with a prospective and retrospective approach, case series and others that address the combination of the following elements (see Table 3):

**Table 3:** Criteria for searching articles

Databases	Language	Criteria
CINAHL	German	Articles covering the period of 2000–2016
IEEExplorer	Spanish	Titles directly related to the theme
LILACS	French	Number of citations and required patent
MEDLINE	Italian	Method used for data processing
PubMed	English	Study groups (men, women, ethnicity, age group)
Web of Science	Portuguese	Number of input variables
Scopus	Mandarin	Exclusion of repeated strategies
Science Direct		

PubMed—US National Library of Medicine National Institute of Health, IEEE Explorer—Digital Library and Science Direct

*LILACS* Latin American and Caribbean Center on Health Sciences Information, *MEDLINE* Medical Literature Analysis and Retrieval System Online, *CINAHL* Cumulative Index to Nursing and Allied Health Literature

- Parameters or diagnostic factors;
- Multidimensional data classification models;
- Image screening as an assistance to the classification of risk groups, no restriction on the minimum number of patients for each study;
- Greater range of study groups (men, women, ethnicity);
- Different approaches to artificial intelligence or intelligent algorithms;

It was not selected: studies with titles not directly related to the research theme and studies that did not contain relevant data related to the research in the abstracts.

## Selection of studies

Using an individual screening of the articles found after searching the electronic database using the mentioned terms, data was extracted with the main and most frequent methods for identifying risk groups of osteoporotic fracture. An organization chart of the methodology adopted to reach the 25 articles chosen for this review is shown in Fig. 1. Primarily, a recognition of all the titles found with the terms descriptors for the search was made. Titles in line with the survey, which emphasized the use of artificial intelligence combined with imaging exams and parameters as risk factors, have undergone initial screening. Subsequently, documents with repeated themes and of smaller number of citations were excluded, leaving those selected for reading the related abstracts. To determine the articles that would have a complete reading, those who did not presented strategies specifically associated with the estimation of individuals with osteoporosis were removed (see Table 4).

**Table 4:** Main articles selected for review

Authors	CIT	Year	Technique	Auxiliary exam	Group	PAC
Koh et al. [14]	465	2001	OSTA	DXA	Women	1983
Kung et al. [50]	68	2002	OSTA	QUS	Men	722
Gregory et al. [13]	41	1999	LVQ, R-PRO	Histological intervention	Men, women	100
Reid et al. [40]	24	2006	Cross-calibration	DEXA	Women	991
Sapthagirivasan et al. [28]	23	2013	SVM, RBF	X-ray	Women	50
Chiu et al. [18]	19	2006	ANN	DEXA	Men, women	1403
Leslie et al. [23]	19	2011	ALG	DEXA	Men, women	4015
Lemineur et al. [19]	16	2007	ANN	DEXA	Women	304
Yoo et al. [27]	16	2013	SVM, RF, ANN, LR	N/A	Women	1674
Wenjia et al. [16]	15	2005	MLP, NNE	DEXA, QUS, PIXI	Women	2934
Hsueh-Wei et al. [46]	13	2013	MFNN	SNP	Women	295
Kavitha et al. [29]	10	2013	SVM	X-ray	Women	100
Juez et al. [25]	9	2010	GA, MLP	DEXA	Women	200
Liu et al. [32]	8	2015	ANN, GA	X-ray	Men, women	725
Jennane et al. [44]	8	2010	ANFIS, SVM, GA, HSGA, LSGA	Microscopia	N/A	18
Tafraouti et al. [30]	7	2014	SVM	X-ray	N/A	39
Lee et al. [20]	6	2008	SVM	DEXA	Women	94
Mantzaris et al. [24]	6	2010	PNNS, LVQ, ANN	DEXA	Men, women	3426
Meneses et al. [21]	5	2008	ANN, MLP, BP	X-ray	Men, women	100
Xinghu et al. [33]	4	2016	ANN	X-ray	Men, women	119
Meneses et al. [22]	3	2009	ANN	X-ray	Men, women	N/A
Harrar et al. [26]	2	2012	ANN, MLP	X-ray	Women	120
Iliou et al. [31]	1	2017	MLP	DEXA	Men, women	1403
Kavitha et al. [45]	1	2012	SVM	X-ray	Women	69
Rizzi et al. [15]	1	2004	MoG, SHEM	CBM	Women	845

*ALG* algorithm, *ANFIS* adaptive neuro fuzzy inference system, *ANN* artificial neural networks, *BP* back-propagation, *CBM* computerized bone mineralometry, *CIT* citations, *DEXA* dual-energy X-ray absorptiometry, *GA* genetic algorithm, *HSGA* hybrid skeleton graph analysis, *LR* logistic regression, *LSGA* line skeleton graph analysis, *LVQ* learning vector quantization, *MFNN* multilayer feedforward neural network, *MLP* Multilayer Perceptron, *MoG* mixture of Gaussian, *NNE* neural net ensemble, *OSTA* Osteoporosis Self-Assessment Tool For Asian, *PAC* number of patients, *PIXI* peripheral dual-energy X-ray absorptiometry, *PNN* probabilistic neural network, *PNNS* probabilistic neural networks, *QUS* quantitative ultrasound, *RBF* radial bias function, *RF* random forests, *R-PRO* resilient propagation, *SNP* single nucleotide polymorphism, *SHEM* splitting hierarchical expectation maximization, *SVM* support vector machines

## Results

From the 90s, the first study [12] was published, reporting on the use of integrated risk factors with artificial neural networks (ANN) aid to identify a subset of high risk of osteoporotic fractures. This study proves that this technique has been shown to be an important element as a screening tool for those who really need BMD assessment and to aid in increasingly effective strategies for fracture prevention. In the first instance it was verified that the ANN performance did not present superior results to the conventional statistical methods for diagnosis of the low bone mineral mass (BMD). This low performance could be related to the inadequate association of the so-called somatic factors or risk factors commonly employed and not with the computational model applied [12].

Another integration with Neural Networks was approached in the late 1990 by Gregory, Junold, Undrill and Aspen, however, there was a great limitation for the process used, since live samples were required—histological sections of the trabecular bone and subsequent digitization of the same—for the implementation of the technique [13].

In 2002, Kung, Ho et al. [50] developed a method for targeting the chinese female population at high risk of fractures for BMD measurement, called the Osteoporosis Risk Assessment Tool for Asians (OSTA), which uses a calculation involving two variables for low and high risk of osteoporosis [14]. The combination of ultrasound examinations with the tool provided a sensitivity of 91% in the identification of individuals at higher risk of osteoporosis.

In 2004, Rizzi et al. [15] proposed a low-cost prevention strategy for estimating the occurrence of osteoporosis, having as parameters a set of individual characteristics correlated with BMD. For this study, the Gauss Mixture (MoG) neural network model, trained by the splitting hierarchical expectation maximization (SHEM) procedure was proposed based on the maximum likelihood approach. It was found from the input characteristics, the most important are age and entry into menopause. Regarding the risk of fracture, the body mass index (BMI) should be considered instead of a resource to provide useful information for the estimation of BMD values [15].

In 2005, Wang et al. [16] developed an intelligent diagnostic support system with integration of heterogeneous models, composed of neural networks and decision trees. It was observed that the studies developed with homogeneous systems did not produce a significant gain of performance. On the other hand, the hybrid sets exceeded the homogeneous generating results with accuracy of 85.79%. These studies also propose the construction of more sets of varied types and sizes, using risk factors selected by estimation techniques. In that same year, Sadatsafavi et al. [17] developed some neural network models from a dataset of 2158 postmenopausal Iranian women in which BMD values were measured by DEXA. They had the most effective results in their work using between 3 and 5 somatic factors (age, weight, postmenopausal years, steroids and estrogen) reaching 86.3% of sensitivity and 72.1% of specificity.

In 2006, Chiu et al. proposed a neural network [18] to identify osteoporosis in the elderly population (mean age 63 years, ranging from 50 to 91 years old, 157 men and 1246 women). With random selection of three groups, being 703 for training group, 350 participants for the selection group and the other 350 in the test set. The most relevant risk factors included demographic characteristics, anthropometric measures and clinical data (gender, age, weight, height, body mass index, postmenopausal status and coffee consumption).

Lemineur et al. [19], in 2007, made use of ANN with Levenberg–Marquardt training algorithm combined with three bone densitometry (BMD) parameters (femoral neck BMD, total body BMD and L2 L4 spine BMD) together with the age of the 304 patients. This study aimed to identify patients with osteoporosis before the occurrence of fracture. The results achieved were 81.66% correctly estimated.

In 2007, 2008 and 2009 the application of neural networks and support vector machine was directed to better evaluation of X-ray or ultrasound images, combining several texture parameters with bone density indicators [20]. Moura Meneses et al. [21], in 2008, ANN was used in bone marrow X-ray tomography for histomorphometric analysis, and a Perceptron Multiplayer model was used. In this case it was proven that ANN is able to distinguish bone mass, thus validating its use for histomorphometric analysis. The same authors in 2009 [22] dealt with the quantitative analysis of the human trabecular bone architecture with application of neural network for the classification of pixels of images, with the objective of analyzing the trabecular structure of enlarged images in high resolution of X-ray. The results showed that, even with the complexity of the trabecular structure, the ANN Feed Foward, with BFGS and OSS learning algorithms, was successful in the recognition of pixels of bone images for quantitative analysis as well as compatibility with the characteristics of the extracted images Of X-ray, which did not occur with the LM training algorithm.

In 2009, the work of Leslie et al. [23] conducted in Canada with 4015 women aged 50 years and over, an osteoporosis surveillance program was implemented as an aid to informing, guiding screening, prevention, and treatment of the disease. The use of algorithms and classification methods was applied to different sets of hospital diagnoses, diagnosis of medical references and prescriptions of medicines for disease treatment. The results obtained achieved a total sensitivity of 93.3%, specificity ranging from 50.8 to 91.4% and 81.2 to 99.1% for osteoporotic discrimination of normal BMD. The algorithms that included drug prescriptions as variables obtained a higher sensitivity compared to those based solely on diagnostic exams.

In 2010, a study was conducted in Mantzaris [24], Greece, with 3426 records, of which the majority were female and only 80 male cases. The aim of this study was to estimate the risk of osteoporosis by applying Probabilistic Neural Networks (PNN) and Learning Vectors Quantification (LVQ), with the best performance being NNP, with a 96.58% efficacy [24]. With the Receiver Operating Characteristic (ROC) analysis we determined the most significant somatic factors to characterize the risk of osteoporosis: age, gender, height and weight. These factors were identified as the most determinant, obtaining the result of the area under the curve (AUC) for each somatic factor. When this factor is higher than 0.5 (see Table 5), it is relevant. Gender however was not very relevant because in this study the number of males was negligible in relation to the females, which we can identify as a limitation in this specific study.

**Table 5:** Area under the curve of diagnostic factor. Mantzaris et al. 2009

Diagnostic factor	Age	Gender	Height	Weight
AUC	0.646	0.503	0.560	0.641

Considering somatic factors, in 2010, Cos Juez et al. [25], developed a mathematical method to identify postmenopausal women with osteoporosis, considering only nutritional factors and lifestyle as influential factors in their diagnosis. The variables obtained through a questionnaire, together with the BMD of the patients calculated by densitometry, were processed using genetic algorithms to identify the most relevant factors in the identification. After this, multilayer perceptron neural networks were used to construct a mathematical model that determined the relationship between the input variables and BMD.

In 2012, Jennane et al. [26] argued through their studies that cortical bone measurement is more appropriate than trabecular bone measurement for osteoporosis screening using Support Vector Machine (SVM) with panoramic dental radiographs of 69 postmenopausal women, BMD performed on the lumbar spine and femoral neck. As results, the accuracy of cortical measurements with mean width and variance was 87% and trabecular measurements with mean length and angle of 65%. Harrar et al. Still in 2012, considered that the Multilayer Perceptron neural network constitutes a reliable platform in the classification, making use of 5 parameters [age, bone mineral content (BMC), bone mineral density, fractal Hurst exponent and texture characteristic of Competition] as input, training and testing the performance network with the k-fold cross validation method. The group used for such study was 60 healthy women aged over 67 years and 60 with cases of osteoporotic fractures aged above 74 years. They completed an osteoporosis risk questionnaire that included: age, personal and family history of fracture, use of smoking, alcohol, menopausal status and use of hormone therapy. After different combinations of parameters, in order to identify the predictive value of each, the best combination of parameters was: age, BMC, BMD and the Hurst Fractal exponent.

A more comprehensive study was conducted in 2013 [27] in a group of 1674 medical records of Korean postmenopausal women, whose goal was to assess the risk of osteoporosis. In this study a comparison was made with several models based on popular machine learning algorithms such as SVM, RF, ANN and LR with four conventional clinical decision instruments: OST, ORAI, SCORE and OSIRIS (see Table 6). The quantitative of 1674 women had osteoporosis at any of the following locations: hip, femoral neck or lumbar spine. The training set was 1000 patients, the remaining (674 patients) were used as a test to predict osteoporosis in postmenopausal women. The SVM predicted risk of osteoporosis with an AUC of 0.827, accuracy of 76.7%, sensitivity of 77.8% and specificity of 76.0% in the total hip, femoral neck or lumbar spine. The somatic factors selected by SVM were age, height, weight, body mass index, duration of menopause, duration of breastfeeding, estrogen therapy, hyperlipidemia, hypertension, osteoarthritis and diabetes mellitus. In this study SVM presented the best results under the receiver operating characteristic curve (ROC) of ANN, LR, OST, ORAI, SCORE and OSIRIS for the training set.

**Table 6:** Selection of variables in machine learning and conventional methods for osteoporosis risk of hip, neck and lumbar

Variables	Machine learning method				Conventional method			
	SVM	RF	ANN	LR	OST	ORAI	SCORE	OSIRIS
Age	o	o	o	o	o	o	o	o
Height	o	o	o					
Weight	o	o	o	o	o	o	o	o
Body mass index	o	o	o					
Waist circumference		o						
Pregnancy		o	o					
Duration of menopause		o	o					
Duration of breastfeeding	o	o	o	o				
Estrogen therapy	o					o	o	o
Hyperlipidemia	o	o		o				
Hypertension	o	o						
Fracture history			o				o	o
Osteoarthritis	o	o	o	o				
Rheumatoid arthritis							o	
Diabetes mellitus	o	o	o	o				

*SVM* support vector machines, *RF* random forests, *ANN* artificial neural networks, *LR* logistic regression, *OST* osteoporosis self-assessment tool, *ORAI* osteoporosis risk assessment instrument; *SCORE* simple calculated osteoporosis risk estimation, *OSIRIS* osteoporosis index of risk

Still in 2013, Sapthagirivasan and Anburajan [28] have proposed the use of the trabecular border on digital hip radiographs for the identification of osteoporosis. Applying a kernel-based SVM, they were able to extract trabecular features from digital hip radiographs, identifying individuals with low BMD. The group involved in this study was 50 women from southern India with no previous history of osteoporotic fracture, of these 28 were used for training and 22 for tests. The best results were achieved with a quintuple cross validation analysis with mean accuracy of 90%. It is important to note that in this study, patients with disorders such as rheumatoid arthritis, endocrine abnormalities, Paget’s disease, hypo and hyperthyroidism, bone integrity and associated malignancy, diabetes, pregnancy and fractures due to severe trauma were excluded. Demographic factors were also considered in this study: age, weight, height and body mass index.

Similar studies were performed that same year by Kavitha et al. [29], who used dental X-rays integrating SVM with an automatic histogram clustering (HAC) algorithm to improve diagnostic accuracy in postmenopausal women with low BMD or Osteoporosis, the study group consisted of 100 women without a record of osteoporosis recruited at the University Hospital of Hiroshima, 60 for training and 40 for tests, aged over 50 years. The results were 93.0, 95.8 and 86.6%, respectively, of precision, sensitivity and specificity In the lumbar spine, 89.0, 96.0 and 84.0% in the femoral neck. To achieve these results, the following inclusion criteria were considered: postmenopausal women, age, and no previous diagnosis of osteoporosis.

In 2014, Tafraouti et al. [30] addressed the integration of SVM with feature extraction based on the Fractional Brownian motion model for osteoporosis identification. To do so, they obtained as base for the study bone X-ray images of 77 patients, acquired at the hospital of Orleans, France. In this process, the original image is subdivided into sub-images, and in each sub-image a rotation is applied at an angle θ, obtaining a signal that is modelled by the fractional Brownian motion. The texture characteristics of each image are obtained by concatenating the characteristics of all its sub-images. These are entered as parameters in four kernel functions (Polynomial, Quadratic, RBF, Linear), with the best result in terms of precision being the linear and polynomial kernel function 95 and 93% respectively.

Several studies were conducted in 2015, Iliou et al. [31], used a set of 589 records extracted from the Greek population, who performed bone and laboratory densitometry examinations, applying a multilayer perceptron classifier with the tenfold cross-validation method. In this study, we considered three and five diagnostic factors for the prediction of osteoporosis risk, classifying them into three categories: normal, osteopenia and osteoporosis. Liu et al. [32] used in their studies a sample of 725 control cases of both genders, of which 228 cases were patients admitted to the National Hospital of the University of Taiwan with the first low trauma hip mass and 215 patients without a fracture of Hip and 282 randomly selected residents. The predictive model in question involves only the elderly over 60 years and focuses on the prevention of risk of hip fracture. All patients were interviewed with the same questionnaire, the data collected were inserted into a database, to identify the most significant variables was applied to the sensitivity analysis and connection weights approach. With this method the input variables were classified according to importance, identifying the 10 main variables (total BMD, home fall, height, BMI, hypertension, fecal incontinence, education), which show a large proportion of contribution to predict fracture of hip. In this study, three-layer retro-propagation ANN models were applied to male and female patients separately. The male model performed better than the female because he had a lower degree of complexity.

Finally, in 2016, Yu et al. [33] they have used as samples the cases 119 hospitalized patients, of which 55 patients with osteoporosis, 64 patients without osteoporosis, mean age 65, 49 male and 79 female. X-ray imaging features were used: increased permeability of the vertebral body, trabecular bone of the vertebral levels disappearing, vertical longitudinal trabecular bone enlargement, spinal deformation and vertebral fracture, added to these are: back or body pain Smoking history, use of glucocorticoids. From the total of 17 parameters, six are imaging characteristics, six are clinical extracted by orthopedists and radiologists and five main complaints. As results the sensitivities were 94.5 and 63.6%, specificity 96.9 and 87.5%.

The main studies of the use of artificial intelligence in the diagnosis of osteoporosis are listed in Table 7, comparing the type of artificial intelligence applied, performance, number of risk factors, number of patients, country and gender of the population considered.

**Table 7:** Main studies in the use of artificial intelligence as an aid to the diagnosis of osteoporosis

Author/article	Conventional method						
	Y	AI	%	VAR	PAC	Country	Gender
Kung et al. [50]	2002	OSTA	91.0	22	722	China	F
Rizzi et al. [15]	2004	MoG	N/A	3	845	Italy	F
Wenjia et al. [16]	2005	Hybrid	85.7	5	2.158	Iran	F
Chiu et al. [18]	2006	ANN	79.2	7	1.403	Taiwan	M/F
Leslie et al. [23]	2009	Algorithm	93.3	5	4.015	Canada	F
Mantzaris et al. [24]	2010	LVQ	96.6	4	3.426	Greece	M/F
Cos Juez et al. [25]	2010	MLP	97.9	10	200	Spain	F
Jennane et al. [44]	2012	SVM	87.0	20	69	Argentina	F
Harrar et al. [26]	2012	MLP	97.0	5	120	France	F
Yoo et al. [27]	2013	SVM	76.7	11	1674	South Korea	F
Anburajan et al. [28]	2013	SVM	90.0	5	50	India	F
Kavitha et al. [29]	2013	SVM	91.8	3	100	Japan	F
Tafraouti et al. [30]	2014	SVM	93.0	16	77	France	M/F
Iliou et al. [31]	2015	MLP	83.0	35	589	Greece	M/F
Liu et al. [32]	2015	MLP	93.0	10	725	Taiwan	M/F
Xinghu et al. [33]	2016	ANN	95.0	17	119	China	M/F

*SVM* support vector machines, *RF* random forests, *ANN* artificial neural networks, *MoG* mixture of Gaussian, *OSTA* Osteoporosis Self-Assessment Tool for Asian, *PNN* probabilistic neural network, *LVQ* learning vector quantization, *MLP* Multilayer Perceptron, *HAC* histogram-based automatic clustering, *M* masculine, *F* feminine, *Y* year, *AI* artificial intelligence, *%* precision, *VAR* amount of variables, *PAC* number of patients

## Discussion

Considering that the pattern of change in BMD with age is reasonably understood as well as there is no independent contribution of BMD to the fracture risk, and it is necessary to associate it with the somatic factors [34–42]. Various methods that make use of the integration of artificial intelligence with risk factors for screening of high risk of osteoporosis or fractures groups. Since screening is intended to guide interventions, the tests should be of high specificity. Based on the literature, it was noted that most of the proposed systems, even using different artificial intelligence approaches, can be very useful for the medical community, provided they are not restricted to specific groups of analysis and have a Spectrum in inclusion of input variables.

Regarding the number of variables Iliou et al. [31], considered 35 parameters for the prediction of osteoporosis risk, to identify the most significant parameters, in this paper, a cross-validation method was used 10 times. With this data set it was possible to categorize individuals into three classes: normal, osteopenia and osteoporosis. In another study [24] it was achieved an excellent performance integrating PNN and LVQ neural networks and for identification of the most significant variables using statistical treatment through ROC analysis, with the most significant factor being age. The limitation in this study is the small number of variables and a small number of males.

A large number of risk factors have been identified witch are directly associated with an increased risk of fracture, [3, 18, 24, 26, 30, 31, 43–50], it has been noted that in some cases its suitability for inclusion in algorithms Evaluation for fracture or osteoporosis prediction was not well validated, it is also worth noting the absence of an international assessment of somatic factors [18, 28].

## Conclusion

It is a fact that osteoporosis is a silent disease that has affected the population worldwide, causing serious injuries, severe pain, loss of mobility in the long term and even premature death [11]. Building a model using artificial intelligence to predict risk groups can ease the burden on health systems, the economy, and society. Developing a comprehensive and effective method could facilitate the use of the low-cost bone densitometry examination, especially for developing countries. We believe that overcoming the challenges addressed here will have a powerful tool to reduce the impact that this disease has on the life quality of the population.

This article presented a direct review of the current literature on the use of artificial intelligence in the identification of groups at risk of osteoporosis or fractures resulting from this disease (see Table 8). The results were summarized. The application of artificial intelligence shows to be adequate for the prognosis of the disease or fracture, since there is no need to provide a diagnostic rule to identify it, but a set of examples that represent the variations of the disease. The resources provided by artificial intelligence have been used successfully in many medical areas such as oncology, urology, surgery and as in the case of this orthopedic article among others. Many classification algorithms and methods have been used for machine learning problems. However, it has been noted that this learning to be successful involves much more than choosing an algorithm and executing it on the data, considering that many learning patterns can present a diversity of parameters, they must be selected properly to obtain better results. The lack of a unified system, with different databases and a grouping of the various attributes and clinical factors to reach a greater range of different populations in the identification of risk groups, among these factors, should be include male participants, upper range of age.

**Table 8:** Input Variables and artificial intelligence that are applied the most in the identification of risk groups of osteoporosis or fractures

Input variables	Artificial intelligence
Abortions or stillbirths Stroke *Height* Arthritis *Physical activities* Hearing Cancer Cataract Alcohol consumption Coffee consumption *Corticoids* Waist circumference Diabetes Difficulty of mobility Heart condition Hepatic disease Chronic Respiratory Disease A Pain when walking Headache or migraine Duration of breastfeeding Education Estrogen therapy Hand hold medium Strength Fracture *Smoking* Pregnancy Hipertension Hyperlipidemia History of falling accidents *Age* BMI—body mass index Urinary incontinence Calcium intake Glucosamine intake Intake of milk Vitamin intake Parkinson’s disease *Menopause* DMO total value Number of children born Occupation Weight MMSE Score *Race* *Gender* Hormone replacement therapy Use of analgesic Use of antidiabetics	ANFIS—adpative neuro-fuzzy inference system *ANN*—*artificial neural network* CNN—condensed nearest neighbor GA—genetic algorithm LVQ—learning vector quantization MFNN—multilayer feedforward neural networks *ML*—*machine learning* NN—nearest neighbor PNN—probabilistic neural network RBF—radial basis function *SVM*—*support vector machine* *LR*—*logistic regression*

Main input variables and artificial intelligence used to identify risk groups for osteoporosis

We believe that the construction of a hybrid system composed of artificial intelligence with a simplified method of examination of bone mineral density, can provide better results, because given the proposal of greater population coverage, the system will have to deal with a high level of data complexity.
